# Comparison of Novel Biomarkers of Insulin Resistance With Homeostasis Model Assessment of Insulin Resistance, Its Correlation to Metabolic Syndrome in South Indian Population and Proposition of Population Specific Cutoffs for These Indices

**DOI:** 10.7759/cureus.33653

**Published:** 2023-01-11

**Authors:** Komal S Jog, Subbiah Eagappan, Raghavan K Santharam, Sridhar Subbiah

**Affiliations:** 1 Department of Endocrinology and Diabetology, Madurai Medical College, Madurai, IND

**Keywords:** lipid accumulation product, tg: hdl ratio, tyg index, homa-ir, metabolic syndrome, insulin resistance

## Abstract

Background

The clustering of risk factors of cardiovascular disease (CVD) in individuals has been defined as Metabolic Syndrome (MetS). The major forerunner of all the components of MetS is Insulin Resistance (IR) which is measured by the Homeostasis Model Assessment of Insulin Resistance (HOMA-IR) and requires the measurement of fasting plasma insulin levels. We attempted to study the performance of lipid-based biochemical markers of IR for the diagnosis of MetS and postulate a population-specific cutoff for these indices in the South Indian population. In this study, we analyzed three lipid-based indices, Triglyceride Glucose index (TyG index), triglyceride: high-Density Lipoprotein (TG:HDL) ratio, and lipid accumulation product (LAP).

Methods

This was a cross-sectional study and included apparently healthy individuals presenting to our hospital for routine Master Health Checkup assessment and apparently healthy population residing in Kallindhiri, a village near Madurai. Based on the anthropometric measurements and blood investigations, Body Mass Index (BMI), Waist hip ratio, Waist height ratio, HOMA-IR, TyG index, TG:HDL ratio, and LAP were calculated. The diagnostic efficacy of these indices was compared against the presence of MetS based on the NCEP ATP III criteria. The receiver operating characteristic (ROC) Curve was performed to discriminate decision levels (cutoffs) of serum markers in early diagnosis of metabolic syndrome. The results were considered significant with a p-value less than 0.05.

Results

We included a total of 192 patients in our study, consisting of 36% (n=70) males and 63% (n=122) females. All the baseline characteristics except height, weight, and HDL cholesterol were comparable between the male and female groups. The values of HOMA-IR, TyG index, TG:HDL ratio, and LAP showed an increasing trend with the BMI. The mean values of HOMA-IR, TyG index, TG:HDL ratio and LAP was significantly higher in patients with MetS than in patients without MetS. Based on the ROC curve plotted for the data, a population-specific cutoff for these indices was computed. Our proposed cutoff for the South Indian population for HOMA-IR is 1.23, for TyG index is 4.65, for TG:HDL ratio is 3.44 in males and 2.6 in females and for LAP is 43.81

Conclusion

The cutoffs for the novel indices of insulin resistance which have been previously studied in Caucasian populations cannot be applied to Indian populations due to distinct ethnic characteristics. The diagnostic accuracy of these novel lipid-based biomarkers of Insulin Resistance is better than the biochemical gold standard of HOMA-IR based on the ROC curve. We propose the usage of these population-specific cutoffs in routine clinical practice for early diagnosis of metabolic syndrome.

## Introduction

It has been observed that risk factors for cardiovascular disease (CVD) tend to cluster together. This clustering of risk factors has been defined as Metabolic Syndrome (MetS) and helps us to identify populations at risk for future CVD and also better understand the pathophysiology of lifestyle disorders and the relationship between various components of MetS [1]. Various studies done in India have shown the prevalence of MetS to be between 33% and 45% [2,3]. These data vary according to the populations being studied. The presence of MetS has been associated with higher cardiovascular events and cardiovascular mortality [4], stroke [5], and nonalcoholic fatty liver disease [6].

Various organizations have proposed definitions for MetS including the World Health Organization (WHO) [7], the European Group for Study of Insulin Resistance (EGIR) [8], and NCEP ATP III [9]. Insulin resistance (IR) affects the downstream signaling pathways of insulin, favoring the MAPK pathway over the Pi3K- Akt pathway, thus leading to a number of undesirable metabolic effects such as diabetes, hypertension, and CVD. Thus, IR is a major forerunner of MetS [1]. The gold standard for the measurement of IR is the hyperinsulinemic-euglycemic clamp method, which is cumbersome and requires technical expertise [10]. An easier, albeit less accurate method of measurement, is the Homeostasis Model Assessment of Insulin Resistance (HOMA-IR) which requires the measurement of fasting plasma Insulin levels, which may not be widely available and inaccurate due to improper standardization [11]. Thus, we attempted to study the performance of lipid-based biochemical markers of IR for the diagnosis of MetS and to propose a population-specific cutoff for these novel indices. In this study, we analyzed three novel lipid-based indices, Triglyceride Glucose index (TyG index), triglyceride: high-density lipoprotein (TG:HDL) ratio, and lipid accumulation product (LAP).

## Materials and methods

Our study was a cross-sectional study. This study was conducted from October 2021 to September 2022. Voluntary response sampling was used to recruit individuals for the study. Ninety-two participants who presented to our tertiary care center as a part of their routine Master Health checkup and 100 participants from the community residing at Kallindhiri, a village in Madurai district were included in the study. The inclusion criteria included an apparently healthy population of more than 18 years of age. Individuals with type 2 diabetes mellitus, systemic hypertension, dyslipidemia, any chronic medical or surgical disease, elderly individuals (>70 years), pregnant females, and individuals not consenting to the study were excluded from the study. A detailed medical history was elicited. Anthropometric measurements including height, weight, waist circumference, hip circumference, and body mass index (BMI) were measured. Height was measured in an erect position with the subjects’ heads in horizontal Frankfurt’s plane to the nearest 0.5 cm using a stadiometer. Weight was measured without shoes and with light clothes to the nearest 0.1 kg using an electronic measuring scale. Waist circumference (WC) was measured midway between the superior border of the iliac crest and the lowermost margin of the ribs at the end of normal expiration. Hip circumference was measured at the level of the greatest posterior extension of the hip. All anthropometric measurements were taken twice, and the mean of the values was considered for analysis. Blood pressure was measured according to the guidelines proposed by the Seventh Report of the Joint National Committee [12]. The participants were instructed to observe an overnight fast of eight hours before presenting for the study. BMI was defined as the ratio of body weight to body height squared, expressed in kg/m^2^. Waist/hip ratio (WHR) was calculated, and a WHR ratio of 0.9 in men and 0.8 in women was taken as a cutoff [13]. Hypertension was defined as systolic blood pressure > 130 mmHg or diastolic blood pressure >85 mmHg [9]. The BMI of the patients was categorized according to the criteria specific to Southeast Asians, with BMI of 23-24.9 kg/m2 defined as overweight and BMI of >25 kg/m2 defined as obese [13].

Blood samples were collected for fasting plasma glucose, fasting plasma insulin levels, fasting serum lipid profile, liver function tests, and kidney function tests. The glucose peroxidase method was used for the estimation of fasting plasma glucose. Fasting insulin levels were done by ECLIA method with ELECSYS kit using COBAS e411 fully automated analyzer. Total cholesterol, HDL-C, and triglycerides were measured using TransAsia Erba XL 1000 automated biochemistry analyzer by direct measurement. LDL-c was derived using the Freidwald formula.

According to the NCEP ATP III definition, MetS was diagnosed if three or more of the following five criteria were met: waist circumference over 90 cm in men or 80 cm in women, blood pressure over 130/85 mmHg, fasting triglyceride (TG) level over 150 mg/dl, fasting high-density lipoprotein (HDL) cholesterol level less than 40 mg/dL (men) or 50 mg/dL (women) and fasting blood sugar over 100 mg/dL [9]. The participants were divided into those with or without MetS according to the NCEP ATP III criteria. HOMA -IR was calculated based on the fasting plasma insulin and fasting plasma glucose values. (HOMA-IR = fasting insulin (μIU/mL) *fasting glucose (mmol/L)/22.5). TyG index (TyG index= Ln [fasting triglycerides (mg/dL) x fasting glucose (mg/dL)]/2), TG:HDL ratio, and LAP [LAP (men)=(Waist circumference-65) x Triglycerides; LAP (women)=(Waist circumference-58) x Triglycerides] were calculated for all participants. The diagnostic efficacy of these indices was compared against the presence of MetS based on the NCEP ATP III criteria.

Statistical analyses

The sample size was calculated using the positive predictive value of the biomarker, as a primary outcome measure. Using data from the reference studies, we estimated a total of 192 subjects, to be recruited with an assumption of 7% precision and a desired 95% confidence interval.

All analyses were conducted using SPSS version 26 (SPSS Inc., Chicago, IL). Descriptive statistics were computed. Shapiro Wilks test was used to assess the normality of the data. Based on the distribution of data, the Student t-test and the Mann-Whitney U test were used judicially to determine the difference of variables between the two groups. The confidence interval was set at 95%. The Chi-square test was used to find gender association. Spearman rank correlation was used to find the direction of association among chosen biomarkers in the study population.

The receiver Operating Characteristic Curve was performed to discriminate decision levels (cut-offs) of serum markers in early diagnosis of metabolic syndrome. The results were considered significant with p value less than 0.05.

## Results

Our cohort consisted of 192 subjects of which 36% (n=70) were male subjects and 63% (n=122) were female subjects. The mean age of the population was 40.47+/- 12.39 years. The mean age of the male population was 39.57+/- 12.65 years and female population was 40.5+/- 12.05 years. In our study, 33% (n=24) males and 27% (n=33) females were diagnosed with metabolic syndrome according to the NCEP ATP III criteria. Other than height, weight, Waist hip ratio and HDL-C, all baseline parameters were comparable in the male and female groups. The baseline characteristics of our cohort have been described in Table 1.

**Table 1:** Baseline characteristics of the study population *median and interquartile range STD DEV- Standard Deviation; BMI- body mass index; FPG- Fasting plasma glucose; LDL- low-density lipoprotein; HDL- high-density lipoprotein; VLDL- very low-density lipoprotein; TG- triglyceride; HOMA-IR- homeostasis model assessment of insulin resistance; TyG Index- triglyceride glucose index; TG:HDL ratio- triglyceride: high density lipoprotein ratio; LAP- lipid accumulation product

	MALE (n=70)	STD DEV	FEMALE(N=122)	STD DEV	P VALUE
Age (years)	39.57	12.697	40.6	12.005	0.692
	Height (centimeters)	167.607	7.9107	153.496	6.6747	<0.001
Weight (kilograms)		64.807	12.4538	54.198	12.0592	<0.001
	BMI (kg/m^2^)	23.0314676	3.9741684	23.001433	4.91433	0.772
Waist circumference (centimeters)		86.843	11.1393	83.934	12.0735	0.1
	Hip Circumference (centimeters)	91.514	10.2275	92.582	9.7953	0.643
Waist hip ratio		0.951903	0.0971434	0.90485	0.0706	<0.001
	Waist height ratio	0.51926	0.071158	0.54744	0.07958	0.015
FPG (mg/dl)		89.849	24.163	86.041	20.6363	0.196
	Fasting Insulin (uIU/L)	6.087	4.310213	6.28381	4.519689	0.847
LDL (mg/dl)		101*	42.349092	91.2*	36.087417	0.283
	HDL (mg/dl)	40.4701429	11.7941004	47.7058	13.7706	<0.001
VLDL (mg/dl)		27.0771429	11.7477	24.3065574	8.7138444	0.117
	TG (mg/dl)	130*	58.739	111*	43.569	0.117
Total Cholesterol (mg/dl)		163.5*	45.087	165*	39.555	0.732
	HOMA-IR	1.4172978	1.17291004	1.3773189	1.1046075	0.862
TyG Index		4.6427288	0.27008151	4.5853364	0.2167308	0.08
	TG:HDL ratio	3.7408534	2.30918804	2.8514483	1.9590732	0.001
LAP		36.3208977	27.3340738	37.0858455	23.530747	0.558

Of the total 192 subjects, 29.5%(n=57) subjects were found to have MetS by the NCEP ATP III criteria. The mean age of subjects without MetS was 37.60 +/-11.54, whereas the mean age of subjects with MetS was 46.23+/-11.88 (p-<0.001). Participants with MetS had a significantly higher age. The participants with MetS also had significantly higher waist height ratio, Systolic BP, Diastolic BP, Fasting Plasma Glucose, Fasting plasma Insulin levels, LDL Cholesterol and TG level. The mean value of calculated HOMA-IR was 2.32+/- 1.27 in patients with MetS, vs a mean value of 0.99+/- 0.781 in patients without MetS. The TyG index, TG:HDL ratio and LAP in participants with MetS was significantly higher than those without MetS. The various parameters in participants with and without MetS have been summarized in Table 2.

**Table 2:** Comparison of various parameters in individuals with and without MetS Std Dev: Standard deviation; MetS- metabolic syndrome; BMI- body mass index; SBP- systolic blood pressure; DBP- diastolic blood pressure; mmHg- millimeters of mercury; FPG- Fasting plasma glucose; LDL- low-density lipoprotein; HDL- high-density lipoprotein; VLDL- very low-density lipoprotein; TG- triglyceride; HOMA-IR- homeostasis model assessment of insulin resistance; TyG Index- triglyceride glucose index; TG:HDL ratio- triglyceride: high density lipoprotein ratio; LAP- lipid accumulation product

Parameter	No MetS (n=135)	Std Dev	MetS (n= 57)	Std Dev	p value
Age (years)	37.6	11.51	46.23	11.88	<0.001
Height (centimeters)	158.62	9.43	158.67	10.88	0.94
Weight (kilograms)	55.15	12.27	64.95	12.86	<0.001
BMI (kg/m^2^)	21.85	4.18	25.75	4.32	<0.001
Waist circumference (Male) (centimeters)	82.43	10.38	95.29	6.93	<0.001
Waist circumference (Female) (centimeters)	80.78	11.07	92.84	9.96	0.002
Hip Circumference (centimeters)	89.5	9.45	98.57	8.02	<0.001
Waist hip ratio (Male)	0.94	0.11	0.97	0.06	0.026
Waist hip ratio (Female)	0.89	0.06	0.93	0.06	0.927
Waist height ratio (Male)	0.49	0.06	0.56	0.04	<0.001
Waist height ratio (Female)	0.52	0.06	0.61	0.06	<0.001
SBP (mmHg)	112.7	12.87	132.14	21.705	<0.001
DBP (mmHg)	74.32	9.57	86.6	13.92	<0.001
FPG (mg/dl)	81.99	12.33	100.298	32.36	<0.001
Fasting Insulin (uIU/L)	4.86	3.64	9.4	4.53	<0.001
LDL (mg/dl)	90.88	36.59	115.73	37.58	<0.001
HDL (Male) (mg/dL)	43.57	11.73	34.51	9.58	0.002
HDL (Female) (mg/dL)	50.04	14.06	41.29	11	0.438
VLDL (mg/dL)	21.58	7.06	34.15	10.37	<0.001
TG (mg/dL)	107.92	35.33	170.79	51.861	<0.001
Total Cholesterol (mg/dL)	160.33	39.196	188.33	40.643	<0.001
HOMA-IR	0.99	0.781	2.32	1.27	<0.001
TyG Index	4.51	0.18	4.83	0.203	<0.001
TG:HDL ratio (Male)	2.77	1.22	5.6	2.74	<0.001
TG:HDL ratio (Female)	2.21	0.78	4.55	2.95	<0.001
LAP	26.2	17.73	61.91	21.25	<0.001

**Figure 1:**
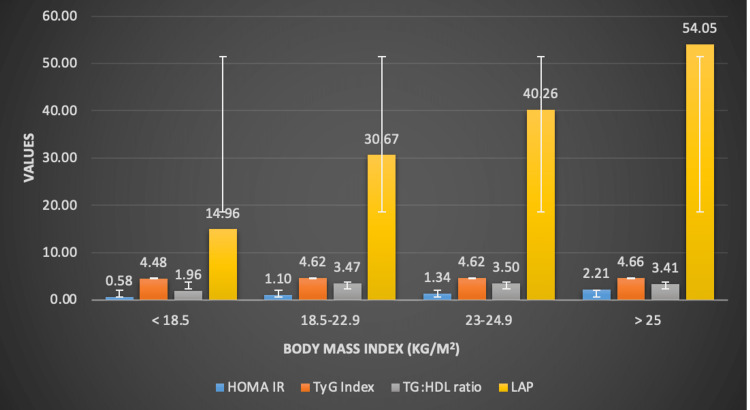
Trend of markers of insulin resistance across various categories of BMI BMI- body mass index; HOMA-IR- Homeostasis model assessment of insulin resistance; TyG index: triglyceride glucose index; TG:HDL ratio: Triglyceride: high density lipoprotein ratio; LAP: lipid accumulation product The x-axis depicts the body mass index (BMI) of the population, which has been subdivided into (i) underweight- BMI: <18.5 kg/m^2^; (ii) normal- BMI: 18.5-22.9 kg/m^2^; (iii) overweight- BMI: 23-24.9 kg/m^2 ^and (iv) obese- BMI: > 25 kg/m^2^

The value of calculated HOMA-IR increased progressively with increasing BMI. The mean HOMA-IR for underweight individuals was 0.58, however in obese individuals with BMI > 25 kg/m^2^, the mean HOMA-IR was 2.21 (p-value-<0.001). The TyG index values increased progressively from underweight to obese individuals. The mean TyG index for underweight individuals was 4.48, however in obese individuals with BMI > 25 kg/m^2^, the mean TyG index was 4.66 (p-value-0.003). There was no significant difference in the mean TyG index of normal BMI individuals and overweight individuals, but significant difference was present between underweight and obese individuals. The TG:HDL ratio values increased progressively from underweight to normal weight to obese individuals in male population. However, in females we observed a lower TG:HDL ratio in obese patients as compared to the overweight and normal weight individuals. The mean TG:HDL ratio for normal weight males was 3.73, whereas in obese males, it was 4.47 (p-value-0.025). In females, the mean TG:HDL ratio in patients with normal BMI was 3.14; however, in obese individuals it was only 2.91. The LAP index values increased progressively from underweight to normal weight to obese individuals in both male and female population. The mean LAP for normal BMI individuals was 14.96, whereas in obese individuals, it was 54.05 (p-value-<0.001). The trend of markers of IR across various categories of BMI are depicted in Figure 1.

The HOMA-IR, TyG index, TG:HDL ratio, and LAP had significant positive correlations with age, weight, waist circumference, hip circumference, waist hip ratio, waist height ratio, systolic blood pressure, diastolic blood pressure, fasting plasma glucose, fasting plasma insulin levels, LDL cholesterol and TG level. HOMA-IR and TG:HDL ratio had significant negative correlation with HDL cholesterol levels. HOMA-IR also had significant positive correlation with the three novel lipid-based indices of IR.

Significantly higher mean HOMA -IR, TyG index, TG:HDL ratio and LAP was seen in obese individuals based on BMI, in participants with increased waist circumference, in those having hypertension, high fasting plasma glucose and high TG levels. Significantly higher HOMA-IR and TG:HDL ratio was also seen in patients having lower HDL-C levels.

The mean HOMA-IR in individuals with MetS was 2.32+/-1.27 whereas in those without MetS it was 0.99+/- 0.781. The mean TyG in individuals with MetS was 4.83+/-0.20 whereas in those without MetS it was 4.51+/- 0.182. The mean TG:HDL ratio in individuals with MetS was 4.99+/-2.89 whereas in those without MetS it was 2.5+/-0.98. The mean LAP in individuals with MetS was 61.91+/-21.59 whereas in those without MetS it was 26.20+/-17.37. All the above data exhibited a significant p-value of < 0.001.

**Figure 2:**
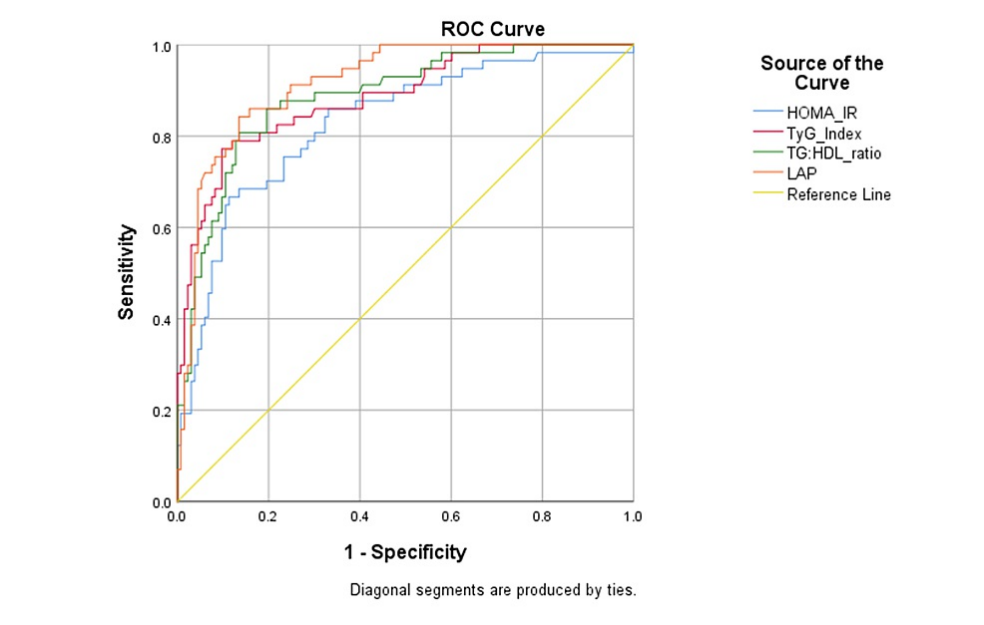
Receiver operating characteristics curve for HOMA-IR and novel lipid-based indices ROC- receiver operating characteristics; HOMA-IR- homeostasis model assessment of insulin resistance, TyG index- triglyceride glucose index; TG:HDL ratio- triglyceride: high density lipoprotein ratio; LAP- lipid accumulation product

Based on the data collected, the optimal cutoff for these indices, specific to the Indian population was obtained by plotting a receiver operative characteristics (ROC) curve. The area under curve (AUC) was highest for LAP index (AUC-0.917) and lowest AUC was observed for HOMA-IR (AUC-0.829). All the indices showed a significant accuracy for diagnosis based on the AUC in ROC curve (p-value-0.001). The ROC curves for all the four indices have been depicted in Figure 2.

Based on the ROC curves, our proposed cutoff for HOMA-IR specific for South Indian population is 1.23. This cutoff value exhibited a sensitivity of 78.9%, specificity of 80%, PPV of 61.4%, negative predictive value (NPV) of 89.9% and a significant Youden’s index of 0.98. Our proposed cutoff specific for TyG index in South Indian population is 4.65. This cutoff value exhibited a sensitivity of 80.7%, specificity of 81%, PPV of 63.2%, NPV of 90.8% and a significant Youden’s index of 0.99. Our proposed cutoff for TG:HDL ratio specific to South Indian population is 3.44 in males and 2.6 in females. The male specific cutoff value exhibited a sensitivity of 79.2%, specificity of 78%, PPV of 59.4%, NPV of 90% and a significant Youden’s index of 0.99. The female specific cutoff value exhibited a sensitivity of 81.8%, specificity of 82%, PPV of 65%, NPV of 91.7% and a significant Youden’s index of 0.99. Based on the ROC Curves, our proposed cutoff for LAP ratio specific to South Indian population was 43.8. This cutoff had a sensitivity of 84.5%, specificity of 85%, PPV of 69.5% and NPV of 92.8% with a significant Youden’s index of 0.98. The cutoffs and their diagnostic efficacy have been summarized in Table 3.

**Table 3:** Receiver operating characteristics curve analyses, sensitivity, specificity, NPV, PPV and Youden’s index for the various biomarkers PPV- positive predictive value; NPV- negative predictive value; HOMA-IR- Homeostasis model assessment of insulin resistance; TyG index: triglyceride glucose index; TG:HDL ratio: Triglyceride: high density lipoprotein ratio; LAP: lipid accumulation product

Index	Optimal cut off value	Sensitivity (%)	Specificity (%)	PPV	NPV	Youden’s Index
HOMA IR	1.23	78.9	80	61.4	89.9	0.98
TyG Index	4.65	80.7	81	63.2	90.8	0.99
TGL/HDL ratio(male)	3.44	79.2	78	59.4	90	0.99
TGL /HDL ratio(female)	2.6	81.8	82	65	91.7	0.99
LAP	43.81	84.5	85	69.5	92.8	0.98

## Discussion

In our study, we studied the diagnostic accuracy of novel lipid markers of insulin resistance for MetS when compared to HOMA-IR, which is a biochemical marker of IR. In the past, various studies have been done to assess the diagnostic efficacy of TyG index, TG:HDL and LAP index and have devised an optimal cutoff for these indices [14-18]. However, there are no studies that have proposed a cutoff for TyG index and TG:HDL ratio in the Indian population. This is important as Southeast Asians have been shown to have different body composition and biochemical parameters such as lower HDL values, due to which cutoffs proposed for Caucasian populations may not be ideal for Southeast Asians [19]. A study done by Ray et al. proposed a cutoff for the LAP index in the South Indian population [14]. 

In our study, 29% (n=57) of the recruited patients were found to have MetS. This finding correlated with the findings of the meta-analyses done by Krishnamoorthy et al. who reported that one in three individuals in the population had MetS [2]. Another study done by Prasad et al. reported the prevalence of MetS to be 24.9 % in males and 42.3% in females [3]. However the quoted study was done in an urban population, thus the increased prevalence in this cohort can be expected. 33% (n=24) of males and 27% (n=33) of females had metabolic syndrome in our cohort. These values reflect true population characteristics, considering that a major portion of our cohort was recruited from rural settings, which form a major part of the South Indian population.

The gold standard for the estimation of insulin resistance is by hyperinsulinemic euglycemic clamp techniques, which were first developed by DeFronzo et al. in 1979 [10]. However, these techniques are cumbersome and need high technical expertise. HOMA-IR is a biochemical marker for the diagnosis of Insulin resistance. However, the EPIRCE study done by Gayaso-Diz et al. found that a blanket cutoff of HOMA-IR cannot be applied to all populations. They devised separate cutoffs for diabetic and non-diabetic populations. Further HOMA-IR did not correlate with cardiovascular mortality in women more than 70 years of age [20]. An Indian study done by Venkatesan et al. showed that HOMA-IR was less reliable when validated against the M-value (steady state value) in a hyperinsulinemic-euglycemic clamp [21]. Thus HOMA-IR may not be a good marker for the prediction of MetS in Asian ethnic populations. Also, there is a lacuna of population-based studies which can propose a specific cutoff for HOMA-IR in Indian populations. In our study, we estimated the optimal cutoff of HOMA-IR for diagnosing IR and MetS in our cohort of mixed rural and urban populations to be 1.23. Another Indian study done by Yashpal et al. in urban adolescents has proposed a cutoff of 2.5 for the prediction of MetS [22]. The higher cutoff in the mentioned study may be because the study was done in the adolescent age group, which has been known to be a phase of physiologically enhanced insulin resistance due to the growth hormone surge during this time. Also, this study included only urban residing individuals, whereas our study was conducted in the rural as well as urban residing population and may be a truer representation of real-world data.

There are various practical difficulties in using an insulin-based assay for population-based screening. These assays are not widely available, expensive, not standardized, and need the maintenance of cold chain and expert trained laboratory staff. Lipid-based indices confer the advantage of being based on a fasting lipid profile and anthropometric measurements. These can be carried out by the primary health care workers at the first point of contact. Lipid assays are widely available and well standardized and require only a fasting blood sample. Thus, they may be a better and more practical tool for the screening of MetS. In our study we examined the performance of three lipid-based indices, TyG index, TG:HDL ratio and the LAP for diagnosis of MetS.

Higher TyG index has been shown to be associated with increased incidence of cardiovascular diseases [23] and incident renal disease [24]. There are no Indian studies which have studied this index. In our study, based on the ROC Curves, our proposed cutoff specific for TyG index in South Indian population was 4.65. This cutoff value exhibited a sensitivity of 80.7%, specificity of 81%, PPV of 63.2%, NPV of 90.8% and a significant Youden’s index of 0.99. Guerrero Romero et al. studied TyG index and proposed a cutoff of 4.68 for diagnosis of IR [17]. Our cutoff closely resembles that of the cutoff proposed by Romero et al. Our study is the first study to estimate the efficacy of TyG index in Indian populations.

Serum TG and HDL are inexpensive tests which can be easily carried out in the community. In a study done by Giannini et al, higher TG/HDL-C ratio was significantly associated with IR mainly in white obese boys and girls and was a useful marker of IR in this population [16]. In yet another study, TG:HDL-C ratio was shown to correlate with the extent of cardiovascular disease, morbidity and mortality [25]. In our study, based on the ROC Curves, our proposed cutoff specific for TG:HDL ratio in South Indian population was 3.44 in males and 2.6 in females. In the MESYAS study TG:HDL ratio values >2.75 in men and >1.65 in women were highly predictive of MetS. These cutoffs had 80% sensitivity and 73% specificity [15]. Our values exhibited similar sensitivity and a higher specificity compared to the MESYAS study. The higher cutoff in our cohort when compared to the cutoffs defined in the MESYAS study may be due to a lower HDL level that have been consistently noted in Asian populations when compared to Caucasian populations [19]. Other studies done in Indian population to study TG:HDL ratio by Kohli et al. and Chauhan et al. found positive correlations between TG:HDL ratio and various parameters such as BMI, body fat and cardiovascular risk factor, but these studies did not propose a diagnostic cutoff for the same [26,27]. Thus, to our knowledge, our study is the first to propose a South Indian specific cutoff for TG:HDL ratio for diagnosis and prediction of MetS. In our study the female population showed a dip in the mean TG:HDL ratio in the obese category of BMI when compared to the overweight category. We attribute this to a small sample size and possible outliers. Studies with larger sample size will give more clarity in this aspect. However, in the male subset of our cohort, a rising trend of TG:HDL ratio with rising BMI was noted.

LAP was first described by Kahn et al. as a better marker than BMI for diagnosing cardiovascular risk and diabetes [18]. Another study done in Taiwanese population also supported the efficacy of this index in the diagnosis of MetS [28]. LAP has been studied as a marker of IR by Ray et al. in South Indian population. In this study, they proposed a cutoff of 38.05 for both genders for the diagnosis of metabolic syndrome [14]. In another study done by Anoop et al. in non-obese Asian Indian males, where LAP was compared to the M value (steady state value) of hyperinsulinemic -euglycemic clamp, a cutoff of >33.4 was determined for the diagnosis of MetS [29]. In our study, based on the ROC Curves, our proposed cutoff specific for LAP in South Indian population was 43.81 which is comparable to the cutoff described previously. Our cutoff had a higher sensitivity but lower specificity than the study done by Ray et al.

When comparing all three novel indices of insulin resistance, we found that LAP had the highest AUC, signifying maximum diagnostic efficacy. TG:HDL ratio and TyG index had similar AUC, however, TyG index had mean value difference of only 0.32 between the MetS and non MetS groups, whereas TG:HDL ratio had a mean value difference of 2.59 between MetS and non MetS. Thus TG:HDL ratio may be a better indicator of MetS among the above two indices.

Our study had a number of strengths. Our's was the first study in India which attempted to propose a South Indian specific cutoff for HOMA-IR and the novel lipid indices of IR like TyG index and TG:HDL ratio. The measurement of Insulin was done by ECLIA method using Roche Cobas e411 appliance, which is reliable and accurate. Our study is a field study which included the populations residing in a nearby village as well as urban populations of Madurai visiting the hospital for screening health program. Thus, our study includes both urban and rural population and thus represents true world population.

Our study had a few limitations as well. Our study was a cross sectional study with a small sample size. Our study had more female representation compared to males. This was because this was a volunteer-based study, and more females were willing to participate in the study. The novel indices were compared against NCEP ATP III criteria and HOMA-IR and not the gold standard, which is hyperinsulinemic euglycemic clamp, due to technical difficulties and non-availability.

## Conclusions

Metabolic syndrome is a progressively increasing burden to our society as it is a precursor to various metabolic disorders such as diabetes, and hypertension and has been shown to have higher cardiovascular mortality and morbidity. Insulin-based markers of IR require a fasting plasma insulin measurement, which may not be widely available and are not well standardized.

Lipid-based indices of insulin resistance such as TyG index, TG:HDL ratio and LAP are novel biomarkers of IR and require only fasting plasma glucose and fasting lipid profile measurements in addition to anthropometric measurements which can be easily conducted in most primary care settings across India. In our study, we have proposed population-specific cutoffs for these novel indices, which may be more apt for our population rather than the cutoffs proposed for Caucasian populations. In our study, we found the ideal cutoff for HOMA-IR in our population to be 1.23. We propose a cutoff of 4.65 for TyG index, 3.44 for TG:HDL ratio in males, 2.6 for the TG:HDL ratio in females, and 43.81 for LAP for the early diagnosis of MetS. We propose that these indices be used in routine clinical practice for early diagnosis of IR and timely interventions for primary prevention.
